# Dietary fibre intake and risk of breast cancer: A systematic review and meta-analysis of epidemiological studies

**DOI:** 10.18632/oncotarget.13140

**Published:** 2016-11-05

**Authors:** Sumei Chen, Yuanyuan Chen, Shenglin Ma, Ruzhen Zheng, Pengjun Zhao, Lidan Zhang, Yuehua Liu, Qingqing Yu, Qinghua Deng, Ke Zhang

**Affiliations:** ^1^ Department of Radiation Oncology, Hangzhou Cancer Hospital, Hangzhou, Zhejiang 310002, China; ^2^ Affiliated Hangzhou First People's Hospital, Hangzhou, Zhejiang 310006, China; ^3^ Affiliated Hangzhou Hospital of Nanjing Medical University, Hangzhou, Zhejiang 310006, China

**Keywords:** breast cancer, dietary fibre, meta-analysis, epidemiology

## Abstract

Current evidence from randomised controlled trials on the effects of dietary fibre intake on breast cancer risk is inconsistent. We conducted a meta-analysis to determine the effectiveness of dietary fibre intake in reducing breast cancer risk. We searched for prospective and case-control studies on dietary fibre intake and breast cancer risk in the English language through March 2016. Twenty-four epidemiologic studies obtained through the PubMed, Embase, Web of Science, and Cochrane Library databases were systematically reviewed. A random-effects model was used to compute the pooled risk estimates by extracting the risk estimate of the highest and lowest reported categories of intake from each study. The meta-analyses showed a 12% decrease in breast cancer risk with dietary fibre intake. The association between dietary fibre intake and breast cancer risk was significant when stratified according to Jadad scores, study types, and menopause status. Dose-response analysis showed that every 10 g/d increment in dietary fibre intake was associated with a 4% reduction in breast cancer risk, and little evidence of publication bias was found. Thus, dietary fibre consumption is significantly associated with a reduced risk of breast cancer, particularly in postmenopausal women.

## INTRODUCTION

Breast carcinoma is the most common carcinoma in women worldwide. Ecological and migrant studies have provided strong evidence that environmental factors, including lifestyle and dietary factors, are related to breast carcinoma risk [1–3]. For example, when women who previously lived in countries with a low risk for breast cancer immigrated to high-risk countries, their risk of developing breast cancer increased [4].

Epidemiological studies have shown conflicting results for the relationship between intake of dietary fibre and breast cancer. Dietary fibre reduce the risk of breast cancer may likely by decreasing the level of estrogen in the blood circulation [5]. Results of the most recent meta-analysis published in 2012, which included 17 publications, supported this hypothesis [6]. However, there have been a lot of recent prospective cohort studies [7–22] on dietary fibre intake and breast cancer, and most of the results show that the association between the two is not significant. Recently, several studies [23–30] observed the vital negative correlation between the two. The protective effect of dietary fibre intake on breast cancer risk have been reported in some cohort studies [15, 20, 27–30], whereas others reported no effect or even a positive association [7–14, 16–19, 21–26]. Due to the difficulties in obtaining precise estimates of intakes of dietary fibre and owing to the limited heterogeneity of fibre intake within geographically confined populations, the results of these analytical epidemiological studies were conflicting.

Thus, our aim was to clarify the two in a large, geographically and culturally heterogeneous cohort. Pre-specific stratified analyses were performed to assess the impact of various study characteristics on outcomes. We also evaluated whether a dose-response relationship existed between the two.

## RESULTS

### Studies and characteristics

**Figure 1:**
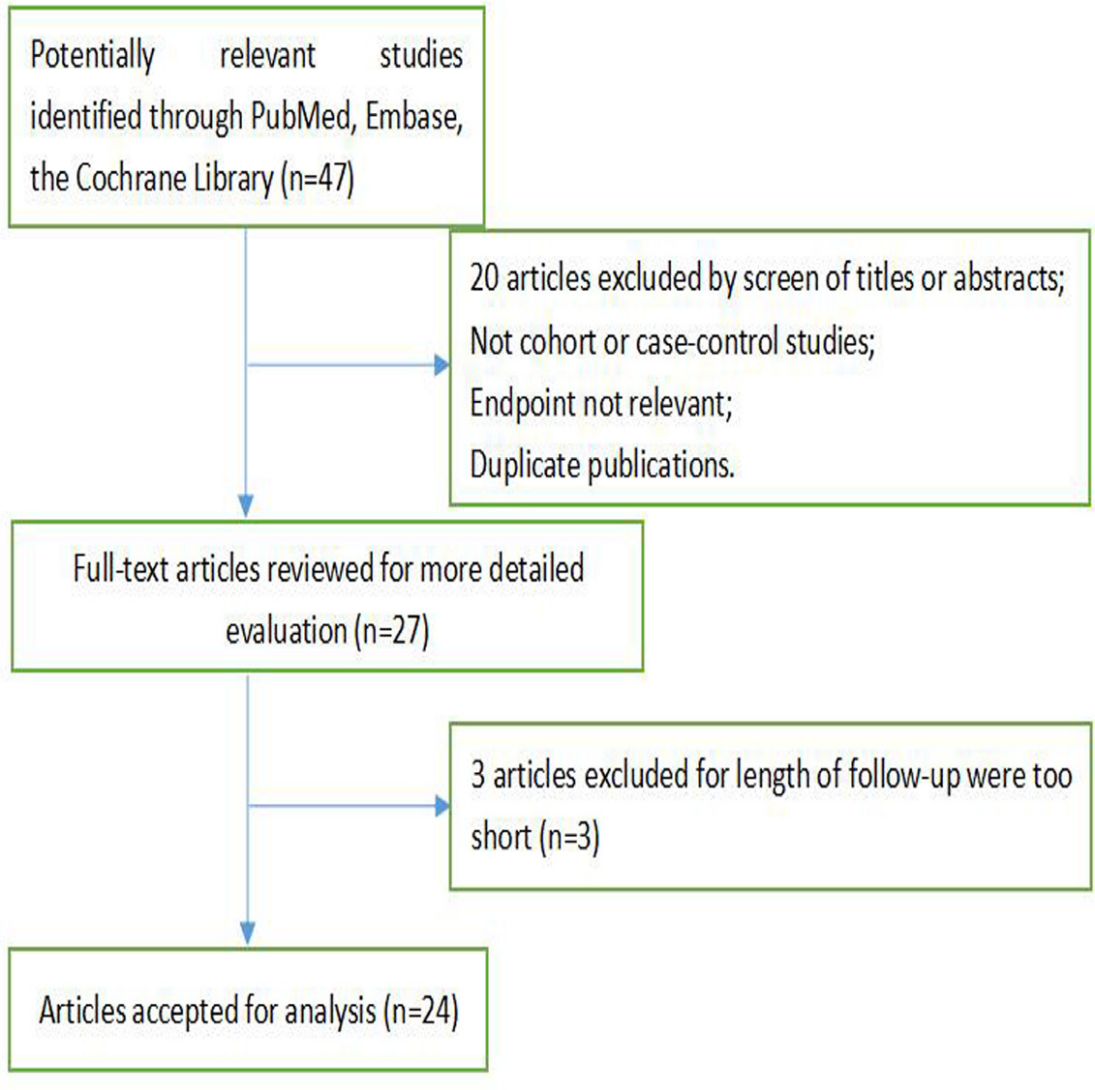
Search strategy and selection of studies

We selected 24 articles from the 47 studies, Which on the relationship between dietary fibre intake and breast cancer (Figure 1). A total of 51,939 cases and 3,662,421 participants were included. All studies included were population-based and their characteristics are listed in Table 1. In this table, a total of eight studies from the United States, two in Canada, twelve in Europe, one in China, and one in Malaysia. The ranges of dietary fibre intake were comparable in most studies, except three studies [11, 18, 28]. Most of the studies have extensively adjusted the potential confounding factors of breast cancer, including smoking; age; body mass index; total energy intake; family history of cancer.

**Table 1:** Characteristics of the studies included in the meta-analysis

Study	Year	Menopause status	Geographic area	Duration	Age range	No. of cases/ sample size	Exposure range (g/d)	Adjusted RR (95% CI)	Adjustment for covariates	Jadad score
Graham et al.	1992	Postmenopause	USA	1980-1987	40–107 y	344/18,586	Q5:>32.7; Q1:<16	1.07 (0.76- 1.51)	Age, education	4
Kushi et al.	1992	Postmenopause	Switzerland	1986-1989	55-69 y	459/34,388	Q5: 27.0; Q1: 14.1	0.99 (0.74-1.31)	Age, age at menopause, age at first birth, family history of breast cancer, body mass index, BMI at age 18 years, waist-to-hip ratio, history of benign breast disease, alcohol intake, total energy intake	4
Verhoeven et al.	1997	Postmenopause	Netherlands	1986–1990	55–69 y	650/62,573	Q5: 34.5; Q1: 16.9	0.83 (0.56- 1.24)	Age; energy intake; alcohol intake; history of benign breast disease; family history of breast cancer; parity; age at menarche, menopause, first birth	3
Terry et al.	2002	Not specified	Canada	1980–2000	40–59 y	2,536/89,835	Q5: >25.8; Q1: <15.2	0.92 (0.78- 1.09)	Age; BMI; smoking; education; physical activity; oral contraceptive use; HRT use; parity; history of benign breast disease; history of breast self-examination; family history of breast cancer; menopausal status, intakes of energy, alcohol, saturated fat.	4
Horn-Ross et al.	2002	Not specified	USA	1995–1996	21-103y	711/111,526	highest;lowest	1.1 (0.8-1.4)	Age, race, daily caloric intake, family history of breast cancer, age at menarche, nulliparity/age at first full-term pregnancy, physical activity, an interaction term for BMI and menopausal status	3
Sieri S et al.	2002	Postmenopause	Italian	1987-1992	41–70 y	56/3,367	Q5:>20.1; Q1: <16.6	0.73 (0.33-1.59)	Energy, parity, place of birth, level of education, total carbohydrates	3
Cho et al.	2003	Premenopause	USA	1991–1999	26–46 y	714/90,655	Q5: 24.8; Q1: 12.5	0.88 (0.67- 1.14)	Age, smoking, height, parity and age at first birth, BMI, age at menarche, family history of breast cancer, history of benign breast disease, oral contraceptive use, menopausal status, alcohol intake, energy intake, animal fat intake	4
Holmes et al.	2004	Not specified	USA	1980–1998	34–59 y	4,092/88,678	Q5: >30; Q1: <10	0.68 (0.43- 1.06)	Age, BMI, total energy intake, alcohol intake, parity and age at first birth, height, family history of breast cancer, history of benign breast disease, age at menarche (y), HRT use, menopausal status	5
Cade et al.	2007	Premenopause	United Kingdom	1995–2004	35–50 y	257/15,951	Q5: >30; Q1:<20	0.48 (0.24- 0.96)	Age, BMI, physical activity, smoking, oral contraceptive use, number of children, alcohol intake, total energy intake	5
Sonestedt et al.	2008	Not specified	Sweden	1991–2004	46–75 y	544/15,773	Q5: 26.0; Q1: 12.0	0.82 (0.61- 1.09)	Age, season of data collection, diet interviewer, method version, total energy, weight, height, education, smoking, physical activity, household activities, alcohol intake, age at menopause, HRT use	5
Suzuki et al.	2008	Postmenopause	Sweden	1987–1997	39–73 y	1,284/51,823	Q5: 29.0; Q1: 16.6	0.85 (0.69- 1.05)	Age, height, BMI, education, parity, menopausal status, oral contraceptive use, HRT use, family history of breast cancer, history of benign breast disease, total energy intake, total fat intake, fruit and vegetable intake, alcohol intake, and age at first birth, menarche, and menopause.	4
Maruti et al.	2008	Not specified	USA	2000-2002	50-76 y	507/28,586	highest; lowest	1.14 (0.82-1.60)	Age, race, mother/sister with breast cancer, mammography within 2 y preceding baseline, history of breast biopsy, age at menarche, age at first birth, age at menopause, combined oestrogen and progestin PMH use, BMI at baseline, past-year alcohol intake, height, and physical activity in past 10 y, total energy intake	4
Lajous et al.	2008	Postmenopause	French	1993-2002	46-61 y	1,812/62,739	Q4:35.2;Q1:15.4	0.99 (0.85,-.16)	Total energy intake	4
Park et al.	2009	Postmenopause	USA	1995–2003	50–71 y	5,461/185,598	Q5: 26.0; Q1: 11.0	0.87 (0.77- 0.98)	Age, race, education, BMI, age at first birth, family history of breast cancer, age at menopause, physical activity, smoking, HRT use, breast biopsy, gynecologic surgery, alcohol intake, total fruit and vegetable intake, total fat intake, total energy intake	5
Wen et al.	2009	Not specified	China	1997–2005	40–70 y	616/74,942	Q5: 16.3; Q1: 7.7	1.09 (0.84- 1.40)	Age, total energy intake, education, BMI, age at first birth, family history of breast cancer, personal history of benign breast disease, physical activity	4
Shikany et al.	2011	Postmenopause	USA	1993-1998	50–79 y	6,115/148,767	Q5:25.1;Q1:8.2	0.93 (0.82-1.07)	Age, ethnicity, education, HRT, Dietary Modification trial randomisation, Calcium and Vitamin D trial randomisation, smoking, alcohol, physical activity, BMI, age at menarche, age at first birth, age at menopause, oral contraceptive use, postmenopausal hormone use, breast cancer in first-degree relative, mammogram within 2 y prior to enrollment, energy intake	5
Zaineddin et al.	2012	Postmenopause	German	2001-2005	50-74 y	2,884/5,509	Q4:24.2;Q1:16.3	0.96 (0.94-1.33)	Menopausal status, BMI, education level, first-degree family history of breast cancer, history of benign breast disease, number of pregnancies (≥28th wk), age at menarche, breastfeeding history, total number of mammograms, smoking habit, alcohol consumption, phytoestrogen supplement use, energy intake, fibre intake	3
Ferrari et al.	2013	Not specified	European	1993-2008	35–70 y	11,576/334,849	Q5:>26.3;Q1:<,17.6	0.95 (0.89-1.01)	Baseline menopausal status, weight, interaction term between weight and baseline menopausal status, height, smoking status, schooling level, physical activity, age at menarche, age at first full-term birth, ever use of a contraceptive pill, ever use of hormones, age at menopause, energy intake, alcohol intake	5
Deschasaux M et al.	2013	Not specified	French	1994-2007	40-54 y	167/4,684	Q4:84.2;Q1:63.9	1.29 (0.66-2.5)	Age (time scale), intervention group, smoking status, educational level, physical activity, height, BMI, number of dietary records, without-alcohol energy intake, alcohol intake, total fat intake, overall healthy dietary pattern, family history of breast cancer, menopausal status at baseline, number of children	4
Li et al.	2013	Not specified	French	1994-1997	30-79 y	557/1,093	Q4:>18.3;Q1:<10.7	1.17 (0.76- 1.80)	Age, race, BMI, age at first menarche, menopausal status, family history of breast cancer, age at first full-term birth, months of lifetime breast feeding, cigarette smoking, alcohol drinking, menopausal status, total energy intake	4
Sulaiman et al.	2014	Not specified	Malaysia	2006-2007	18-80 y	382/1,040	Q4:25;Q1:16	0.31 (0.12-0.79)	Age, ethnicity, marital status, education, working status, household income, age of menarche, age of menopause, pregnancy history, age at first childbirth, number of live birth, history of breastfeeding, duration of breastfeeding, history of oral contraceptive usage, history of HRT usage, smoking habits, alcohol consumption, physical activity level, family history of breast cancer, BMI energy intake	3
Bradbury et al.	2014	Not specified	European	1992-2000	Null	4,517/>500,000	highest compared with lowest quintile	0.93 (0.87- 0.99)	Total energy intake	5
Liu et al.	2014	Postmenopause	Canada	2002-2003	25–74 y	2,865/6,164	Q5: >13.4; Q1:<5.2	0.66 (0.55-0.78)	Total dietary fibre intake 2 y before study enrollment; intakes of vegetable protein and vegetable fat during adolescence further adjusted for vegetable intake 2 y before study enrollment	5
Farvid et al.	2016	Premenopause	USA	1998-2011	27-44 y	2,833/1,725,295	Q5:27.5;Q1:11.1	0.81 (0.72-0.91)	Adolescent alcohol intake, adolescent energy intake	3

USA: the United States of America, BMI: body mass index, CI: confidence interval, HRT: Hormone-replacement therapy, PMH: postmenopausal hormone, RR: relative risk, Null: not given; Q: quintile

### Main results

**Figure 2:**
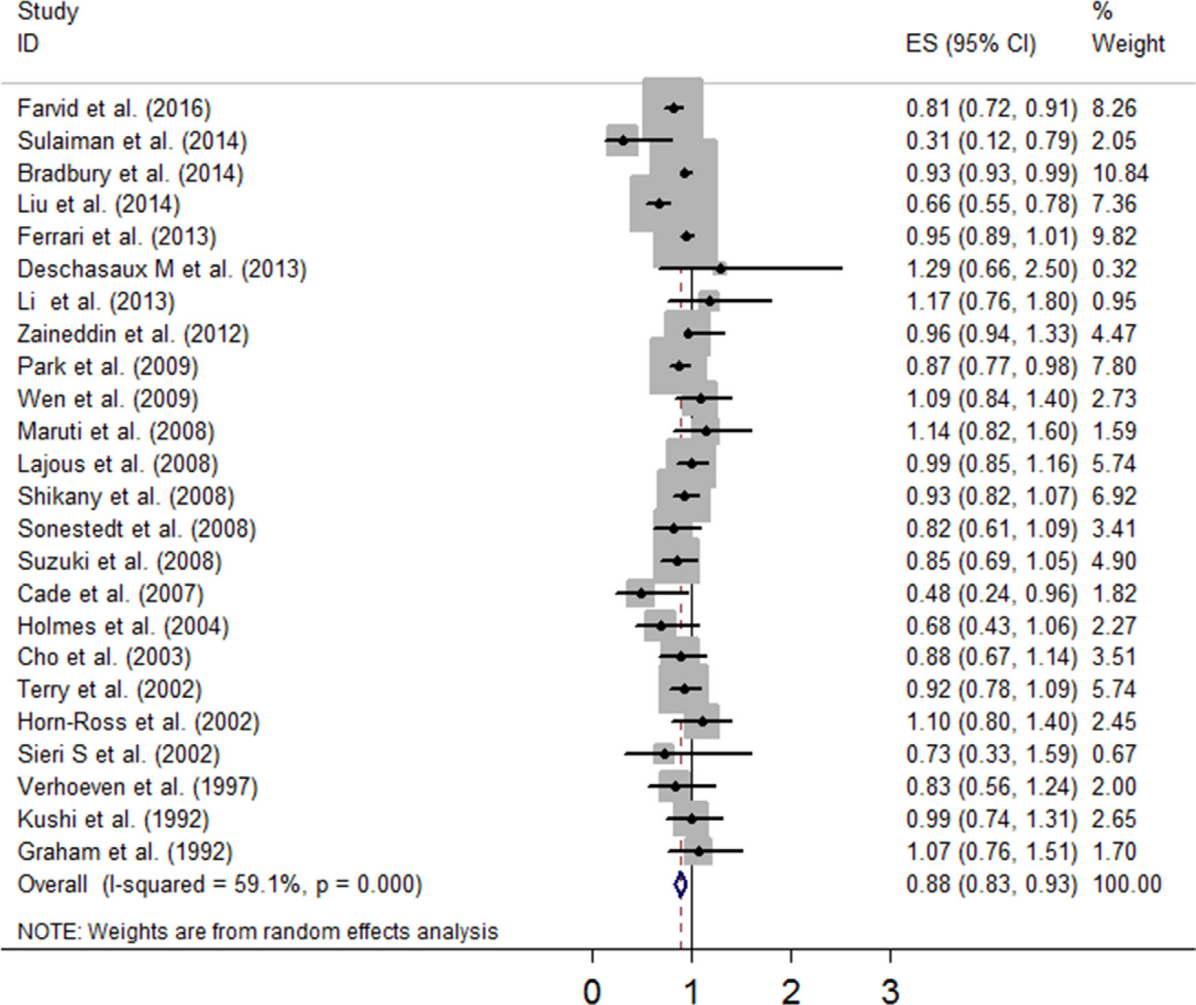
Forest plot of studies evaluating the association between dietary fibre intake and risk of breast cancer

**Figure 3:**
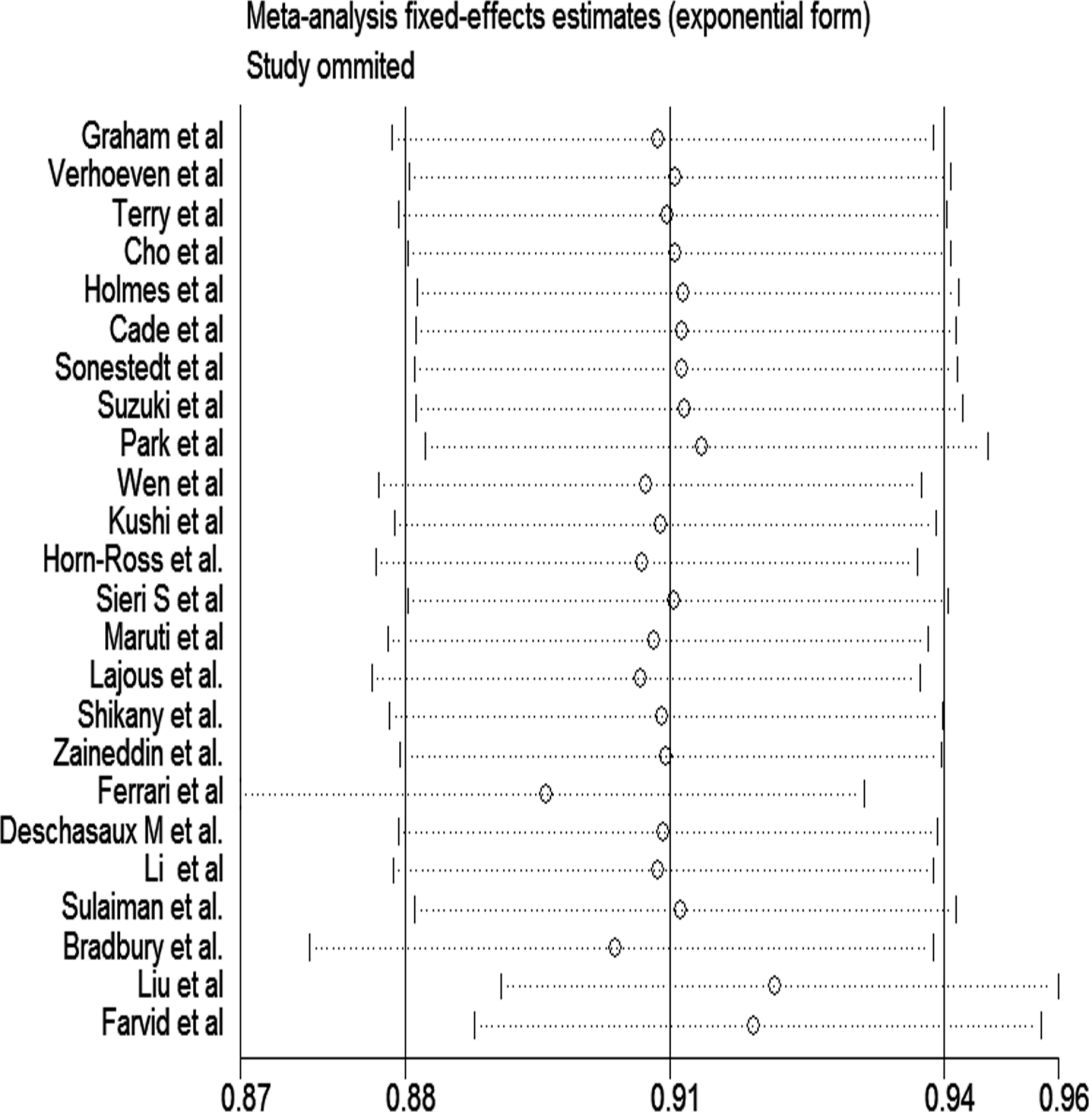
Sensitivity analysis of dietary fibre intake and risk of breast cancer

**Figure 4:**
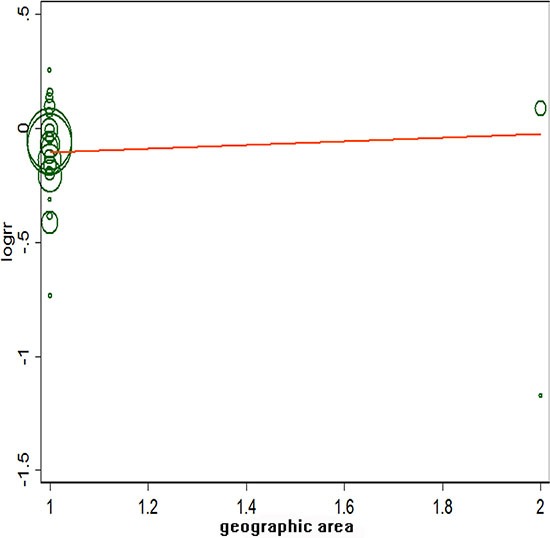
Meta regulation of geographic area and risk of breast cancer

Figure 2 showed a positive association between dietary fibre intake and risk of breast cancer of the twenty-four selected studies. Overall, for the final RR was 0.88 (95% CI: 0.83–0.93). The heterogeneity across studies is acceptable (*P* = 0, *I*^2^ = 59.1%). Figure 3 certified that the publication dates were similar. The meta regulation test showed that geographical area was associated with ~23.4% heterogeneity reduction across the studies (Figure 4).

### Subgroup and sensitivity analyses

Table 2 shows the results of the subgroup analyses according to menopausal status, Jadad score, influence factors, study types, and geographic region. There were no evidence of heterogeneity in case-control studies, postmenopausal women, and with a Jadad score of 3/5 among all influence factors and geographic regions. The association between dietary fibre intake and breast cancer risk will not alter for geographic area, duration of follow-up, and menopausal status. Sensitivity analysis was to investigate a single study on the overall risk assessment. All combined RRs were statistically significant and similar to one another, and the geographical area was associated with an approximately 48.6% reduction in heterogeneity across studies.

**Table 2:** Stratified analysis of breast cancer risk in relation to dietary fibre intake according to study characteristics

Group	No. of studies	RR (95% CI)	*P*_heterogeneity_	*I*_2_(%)
**Menopausal status**				
Premenopause	3	0.78 (0.62–0.94)	0.172	43.2
Postmenopause	10	0.88 (0.79–0.97)	0.027	52.1
Not specified	11	0.92 (0.84–0.99)	0.016	54.2
**Jadad score**				
3	6	0.82(0.64–0.99)	0.014	65.1
4	10	0.96 (0.88, 1.03)	0.796	0
5	8	0.85 (0.77,0.93)	0	76.9
**IF**				
>3	21	0.89 (0.83-0.94)	0.002	54.1
3	3	0.77 (0.48, 1.07)	0.002	83.8
**Study types**				
Cohort	20	0.91 (0.87, 0.95)	0.210	19.6
Case-control	4	0.75 (0.47–1.02)	0.001	80.6
**Geographic region**				
Developed countries	22	0.89 (0.84, 0.94)	0.004	50.4
Developing countries	2	0.71 (0.66–1.47)	0	91.8

IF, influence factor; No., Number; RR, relative risk; CI, confidence interval.

### Dose-response analysis

Since the data required in the three studies are not provided [11, 18, 28], we performed a dose response analysis for the rest of the study. Overall, dietary fibre intake increased by 10 g/D, with a 4% reduction in breast cancer risk (RR: 0.96; 95% CI: 0.92–0.98; *P* = 0.002), and the heterogeneity was not observed (*P* = 0.43).

### Publication bias

**Figure 5:**
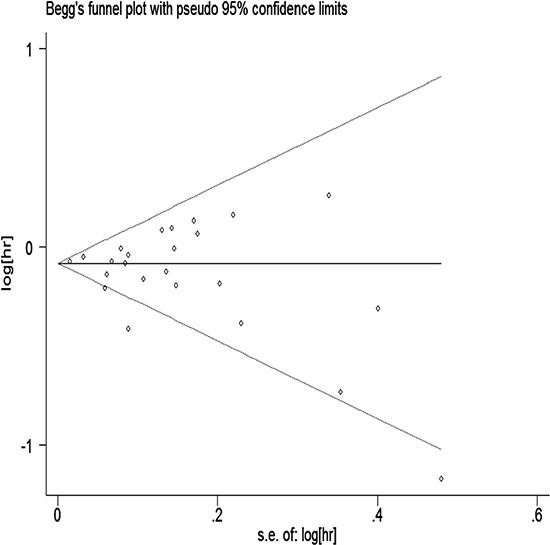
Begger's funnel plot assessing publication bias among studies

**Figure 6:**
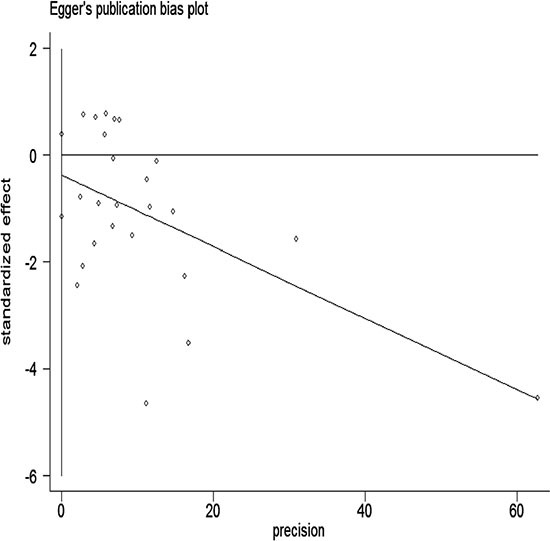
Egger's funnel plot assessing publication bias among studies

We performed Begg's test and Egger's test in all studies. They both indicated little evidence of publication bias (*P* > 0.05; Figures 5 and 6).

## DISCUSSION

This manuscript aimed to clarify the association between dietary fibre intake and the risk of breast cancer. Our analyses showed a protective association between dietary fibre intake and breast cancer risk, and the risk could reduce by 12% on our result. Furthermore, an increment of every 10 g/d increment of dietary fibre intake was associated with risk reduction of 4%.

The size of heterogeneity may be the focus of Meta analysis. In this study, the heterogeneity can be partially explained by these facts: all included studies were conducted in Western countries except two [21, 27], there are many similarities in the population, such as genetic background, dietary patterns, lifestyle. One possible explanation for the heterogeneity is that the source of dietary fibre in each article was different. Moreover, we cannot rule out other good lifestyle habits of participants, which could prevent breast cancer.

The results from our subgroup and sensitivity analyses were strong, which was not significant associated with the geographic region, influence factor of studies, Jadad score, study types, or menopause status. A significant negative correlation between dietary fibre intake and breast cancer risk was observed in many subgroups, for example, postmenopause women, those with a Jadad score of 3 and 5, developed countries, developing countries, influence factor over 3 or lower than 3, and in case-control studies. Estrogen may have different metabolic pathways in premenopausal and postmenopausal women [31], no link was observed in the premenopausal women, probably due to a small number of studies (*n* = 3) included in this meta-analysis, yielding a low statistical power [32, 33]. In addition, several studies are different from some aspects of others. For instance, the French cohort [19], which started in 1993, only adjusted for total energy intake. On the other hand, the US cohort [7], which adjustment factors was too few, and the Italian cohort [12], which started in 1987, the range of the highest and lowest dietary fibre intake was relatively narrow (20.1 g/d vs. 16.6 g/d, respectively).

The following mechanisms may explain the inverse association between dietary fibre intake and breast cancer risk. Dietary fibre is composed of a variety of monomers forming carbohydrate polymers, which cannot be digested and absorbed by the small intestinal in the human body. These polymers mainly include cellulose, hemicellulose, pectin, hydrophilic colloid substances, lignin from plant cell walls, and other components that cannot be degraded by human digestive enzymes [34, 35]. These components of dietary fibre not only absorb and retain moisture, but more importantly, combine with harmful and carcinogenic substances in the gut and promote their discharge and decomposition [36]. Further, dietary fibre can promote the growth of probiotics and inhibit the growth of pathogenic bacteria, thereby inhibiting production of carcinogens and promoting their decomposition in the intestine. It also improves the phagocytosis of macrophages, blocks nitrosamine synthesis, and reduces oestrogen levels. Dietary fibre can also promote the short-chain fatty acids (SCFAs) produced by bacterial fermentation in the colon. Studies have shown that SCFAs are closely associated with tumour development. SCFAs can inhibit the anti-apoptotic gene bcl-2 and promote expression of the pro-apoptotic gene bax. As such, dietary fibre promotes cell apoptosis, thereby preventing development of cancer [37]. Dietary fibre has important physiological functions such as adsorption of ions and organic compounds as well as free oestrogen formed by human intestinal microbial enzymes, which may reduce the risk of breast cancer. Dietary fibre may also have the function of controling the insulin-like growth factors and insulin resistance, thereby protecting against type 2 diabetes mellitus [38]. These factors may also affect the occurrence of breast cancer [39, 40]. Food rich in dietary fibre is known to have a protective effect on breast cancer, and with an increase in consumption of dietary fibre, its protective effect is enhanced. This may be because the fibre can reduce the level of female mammary hormones in the blood, which reduces the occurrence of breast carcinoma [41]. Currently, evidence on dietary fibre is limited, and therefore, future studies concerning the factors in dietary fibre that influence the risk of breast cancer are needed.

Our study has strengths. Our meta-analysis involved enlarged number of studies and participants to date. Besides, we conducted the dose-response analysis to quantify the relationships between dietary fibre intakes and risk of breast cancer.

Despite our important findings, there were a few potential limitations that should be noted. First, unmeasured or uncontrolled confounders should always be accounted for in the selected studies (for example, genic mutation, pecuniary condition). Although most of the studies have an extensive coverage of adjustment, while other residual confounding factors should be considered for further investigation. Second, random errors in dietary fibre intake may have an effect on the outcome, which is inevitable. Third, due to the current articles were mainly based on data from Europe and the United States, our hierarchical analysis shows that geographical changes in a large extent contributed to the substantial heterogeneity.

Our results have a guiding significance in breast cancer. Worldwide, the most common cancer in women remains breast carcinoma, after years of medical development, the 5 year survival rate of breast cancer is not high. Moreover, in some European regions and the United States the intake of dietary fibre are about 22 g/day, this is far below the recommended intake [42]. The existing data are inconsistent from selected studies. Therefore, the clarification of this issue in this study was both important and timely.

In summary, this meta-analysis of epidemiological studies demonstrates that dietary fibre intake was associated with a significant dose response relationship between breast cancer risk and breast cancer risk. Due to the large burden and high incidence of breast cancer, measures for prevention of breast cancer are necessary, and increasing the dietary fibre intake in the diet of the general population may have important prevention of breast cancer.

## MATERIALS AND METHODS

### Literature search

We performed a systematic search for relevant publications by searching PubMed (http://www.ncbi.nlm.nih.gov/pubmed/), Embase (http://www.embase.com/), Web of Science (http://wokinfo.com/), and the Cochrane Library (http://www.thecochrane library.com/) databases through March 2016. Both case-control and cohort studies published in the English language were searched for using the search terms ‘dietary fibre OR fibre OR fibre’ and ‘breast cancer OR breast neoplasms’. In addition, we manually searched through the reference lists of original articles, recent reviews, and meta-analyses. However, we did not contact the authors for the unpublished studies.

### Study selection

Studies fulfilling the following criteria were included in our analysis: (1) the exposure of interest was intake of total dietary fibre; (2) the endpoint was breast cancer incidence; (3) the study design was prospective, i.e. cohort or case-control study; and (4) the relative risk (RR) and corresponding 95% confidence interval (CI) values were reported for the highest and lowest categories of dietary fibre intake. If the same population was considered in more than one study, we included the study with the maximally adjusted model.

### Data extraction and quality assessment

We recorded the following study characteristics: (1) last name of the first author; (2) publication year; (3) menopause status; (4) geographic area; (5) duration; (6) age range; (7) no. of cases/sample size; (8) exposure range (total dietary fibre intake expressed uniformly as g/d); (9) RR from the most fully adjusted model for the highest and lowest dietary fibre intake and the corresponding 95% CI; (10) adjustment for potential confounders in multivariate analysis; and (11) the Jadad score, a scale used to measure the quality of each trial, which ranges from 0 to 5 according to the descriptions of randomisation, blinding, and reporting of participant withdrawals [43].

### Statistical analyses

Incidence rate and hazard ratios ratios were deemed as RR. In highest and lowest categories of dietary fibre intake in meta-analyses, the RR estimate from each study was weighted by the inverse of the variance to calculate RR and 95% CI. We calculated the *Q* and *I*^2^ statistics to examine statistical heterogeneity across studies [44]. A random-effects models [45] was used to calculate the combined RR. Results from the random-effects model, which considered both within- and between-study variations [45], were noted. A sensitivity analysis was conducted using random-effects models to evaluate the robustness of the results.

Pre-specified subgroup analyses were performed according to geographic regions, influence factor of the studies, study types, Jadad scores, and menopause status to assess the potential effect modification of these variables on outcomes. We conducted a sensitivity analysis to investigate the influence of a single study on the overall risk estimate by omitting one study in each turn. In addition, we performed meta-regression analyses to explore the possible explanations for heterogeneity among trials.

To quantify the dose-response relationship between dietary fibre intakes and breast cancer incidence, we conducted a dose-response analysis based on data for different categories of the average dietary fibre dose [46]. Studies were excluded if the required data were not reported or could not be estimated.

To determine the presence of any publication bias, we performed both Egger's and Begg's tests [47], and inspected the funnel plots. All analyses were performed using STATA version 13.0. Values of *P* < 0.05 were considered statistically significant, unless otherwise specified.
